# How stress mediators can cumulatively contribute to Alzheimer’s disease An allostatic load approach

**DOI:** 10.1590/1980-57642018dn13-010002

**Published:** 2019

**Authors:** Tatiane Martins Matos, Juliana Nery De Souza-Talarico

**Affiliations:** 1 Nurse, Master of Science from the School of Nursing, University of São Paulo (EE-USP), SP, Brazil.; 2 Professor at the Department of Medical-Surgical Nursing, School of Nursing, University of São Paulo (EE-USP), SP, Brazil. PhD In the Area of Neurobiology of Stress and Cognition.

**Keywords:** stress, Alzheimer’s disease, allostatic load, cognitive decline, memory, estresse, doença de Alzheimer, carga alostática, declínio cognitivo, memória

## Abstract

Allostatic load is defined as the frequent activation of the neuroendocrine, immunological, metabolic and cardiovascular systems, which makes individuals more susceptible to stress-related health problems. According to this model, physiological dysregulations start to emerge decades before diseases manifest. Consequently, stress research has shifted its attention to anticipating the degree of this dysregulation to better understand the impact of stress hormones and other biomarkers on disease progression. In view of the growing number of studies that demonstrate the influence of modifiable risk factors on cognitive decline, in addition to the effects of chronic stress mediators, the objective of the present review was to present an overview of the development of cognitive changes based on studies on stress and its mediators.

## RESUMO.

A carga alostática é definida como a ativação frequente dos sistemas neuroendócrino, imunológico, metabólico e cardiovascular, o que torna os indivíduos mais suscetíveis a problemas de saúde relacionados ao estresse. Segundo este modelo, as desregulações fisiológicas começam a surgir décadas antes das doenças se manifestarem. Consequentemente, a pesquisa de estresse tem desviado sua atenção para antecipar o grau de desregulação para entender melhor o impacto dos hormônios do estresse e outros biomarcadores na progressão da doença. Tendo em vista o crescente número de estudos que demonstram a influência de fatores de risco modificáveis no declínio cognitivo, além dos efeitos dos mediadores crônicos do estresse, o objetivo da presente revisão foi apresentar uma visão geral do desenvolvimento de alterações cognitivas a partir de estudos sobre o estresse e seus mediadores.

Although various factors are associated with cognitive decline during aging, it is unclear which factors trigger the neurodegenerative process in cases of dementia. Chronic exposure to stress throughout the lifespan has been the focus of many studies because of the similarities between the biological mechanisms involved in chronic stress and the pathophysiology of Alzheimer’s disease (AD), one of the leading causes of dementia worldwide. There is consistent evidence showing that cortisol, the main stress mediator, is associated with both cognitive decline and AD. However, it remains unclear whether the altered cortisol is the cause or consequence of neurodegeneration. Beta-amyloid (Aβ) protein deposition, neuroinflammation, hyperphosphorylation of tau protein, changes in glucose metabolism and insulin signaling are some of the effects of primary mediators of stress that cumulatively can cause decreases in the synaptic network and neuroplasticity, hippocampal and cortical atrophy, and consequently, cognitive decline. This “wear and tear” of chronic stress known as allostatic load is related to the prolonged and sustained exposure to the primary stress mediators that progressively damage brain structures associated with cognitive functions, increasing the risk of disease. This review will explain the similarities between the mechanisms that trigger AD and the effects of stress mediators in the brain to support the hypothesis that, through the allostatic load process, chronic stress during lifespan may seed the vulnerability for developing cognitive impairment later in life.

## ALZHEIMER’S DISEASE: NEUROPATHOLOGY AND ASSOCIATED FACTORS

Alzheimer’s disease is the most common form of dementia and is responsible for approximately 60% to 80% of the cases of the disease worldwide.^1^ The syndrome, which affects approximately 35 million people worldwide, is characterized by gradual and progressive cognitive impairment, including memory decline, impaired reasoning and judgment and slow processing speed allied with executive dysfunction, behavioral and functional decline.^2^^,^ 3 AD leads to the death of nerve cells, gradually causing atrophy in different brain regions which, over time, affects almost all of its functions. The exact neuropathological mechanism by which this neurodegenerative process unfolds is still being studied. It has been established that cognitive and behavioral AD symptoms are correlated with the accumulation of amyloid plaques in the extracellular environment and with intracellular neurofibrillary tangles, which destroy synapses essential to learning, memory, planning and decision making.^4^^-^ 6 Amyloid plaque formation is due to the accumulation of Aβ peptide, which, in turn, is caused by a change in the amyloid precursor protein (APP) cleavage process.^7^ APP is a transmembrane protein which normally undergoes either neuroprotective or amyloidogenic cleavage. Through the action of the β-secretase enzyme, APP is transformed into Aβ peptide. In early-onset familial AD, accumulation of Aβ peptide is related to genetic insults in APP and mutations in presenilin genes (PSEN). After age, the presence of the E4 allele of the gene that codes apolipoprotein E (APOE-ε4) represents the largest risk factor for Aβ accumulation in late-onset AD. In addition, epigenetic changes and environmental risks also contribute to the accumulation of toxic species of Aβ in late-onset AD.^4^

When hyperphosphorylation of tau protein forms neurofibrillary tangles within the cells, cell morphology is altered (microtubule disassembly), disrupting axoplasmic flow and inducing neuronal dysfunction and neurodegeneration.^7^^,^ 8 One of the hypotheses for this hyperphosphorylation is that, when combined with neuroinflammation and oxidative stress, Aβ accumulation causes dysregulation in calcium channels and hyperactivation of kinases, leading to tau hyperphosphorylation.^4^^,^ 9 ^,^ 10

Various studies with animals and humans have shown that neuroinflammation, mediated by microglial activation, plays an important role in the amyloidogenic process (for a review, see Hommet et al., 2014).^11^ Although microglial activation is beneficial in AD because it facilitates the elimination of Aβ peptide,^12^^-^ 15 it may also damage brain tissue due to the release of proinflammatory mediators, such as IL-1β, IL-6, and TNFα.^11^ Neuroinflammation, in turn, leads to oxidative stress, cell damage and death in structures essential for cognitive abilities and, consequently, to cognitive decline.^11^ However, the exact factor that contributes to the activation of this neuroinflammation process is still unclear. Although genetic elements such as APOE alleleε4 are significant AD risk factors, other factors represent risk conditions for the disease. Similarities between the effects of chronic stress and the neuropathological mechanisms involved in the development of AD have reinforced the hypothesis that chronic stress is an important risk factor for cognitive decline during the aging process and for the conversion of mild cognitive impairment (MCI) to AD.

The present article reports evidence that supports the hypothesis that chronic stress may be one of these “other factors” involved in the AD neurodegenerative process.

## ACUTE AND CHRONIC STRESS: PRIMARY MEDIATORS, SIDE EFFECTS, AND ILLNESS

Since its introduction in medical sciences in the 1930s, the concept of stress has evolved, especially because of advances in the field of neuroscience. Stress is a natural and adaptive reaction to challenging or threatening situations and is, therefore, beneficial and necessary for the body to continue functioning. In the short term, various biological, cognitive and behavioral modifications take place so that individuals can adapt to stressful stimuli (acute stress response).^16^ However, stress responses maintained for prolonged or repetitive periods (chronic stress response) can affect the operation of the organism’s adaptive biological systems, causing illness.^16^

Stress response starts with the perception that something threatening or challenging is occurring, i.e., when someone is exposed to a stressful event. Stressors can be classified into two types: real or absolute, i.e., those that are undeniably life-threatening (for example: natural catastrophes, or situations of violence, such as a kidnapping or assault) and relative stressors, where the perception of threat or challenge essentially depends on interpretation (for example: new or unpredictable situations, with little control or social judgment). Unlike real stressors, not everyone responds the same way or with the same intensity to relative stressors. New or unpredictable stressors may represent a challenge or threat to some people, whereas for others, stress occurs in situations over which they have little control or in which they are being judged by others. Whether real or relative, exposure to stressors activates the sympathoadrenal system (SAM), which stimulates catecholamine (adrenaline and noradrenaline) secretion by the adrenal medulla and results in cardiovascular changes (increased heart rate and vasoconstriction), metabolic changes (increased oxygen supply and glucose bioavailability), immunological changes (increased anti-inflammatory factors and coagulation) and cognitive changes (activation of attention and memory for decision-making). The hypothalamic-pituitary-adrenal (HPA) axis is then activated, which stimulates the neurons of the paraventricular nucleus of the hypothalamus to secrete corticotropin-releasing hormone (CRH). CRH acts on the anterior pituitary to release adrenocorticotropic hormones (ACTH), responsible for stimulating the cortex of the adrenal gland to synthesize and release glucocorticoids (corticosterone in animals and cortisol in humans).^16^^-^ 18 These glucocorticoids then cross the blood-brain barrier, binding to specific receptors - mineralocorticoids (MR) and glucocorticoids (GR) located in brain structures intrinsically related to memory and attention. The interaction of glucocorticoids with these receptors exerts negative feedback, acting on the hypothalamus, which reduces CRH synthesis and ACTH secretion in the anterior pituitary and regulates plasma cortisol concentration, restoring it to concentrations prior to exposure to the stressor.^18^^-^ 20

Activation of the SAM and HPA axes modifies the operational parameters of stress response target systems (cardiovascular, immunological, neuroendocrine and metabolic systems), so that, in the short term, energy is mobilized in the form of glucose to prepare the organism for a fight-or-flight response.^21^ These adaptive modifications, called the allostatic response or allostase, occur through the action of primary mediators (glucocorticoids, dehydroepiandrosterone [DHEA], catecholamines, glucose, and pro- and anti-inflammatory cytokines [IL-6 and TNF-α]) that act on the mitochondrial DNA of target system cells to enhance the ability of the mitochondria to produce energy and, consequently, boost cellular energetic capacity for the fight-or-flight response.^21^^,^ 22 Glucocorticoids also modulate the mitochondrial function, increasing membrane potential, Ca^2+^ signaling, and resistance to apoptosis.^23^

Overall, in acute situations, these primary mediators (i.e. cortisol, glucose, IL-6, DHEA and catecholamines) seek to ensure the organism’s best possible adaptation to the demands imposed by stressors. However, in chronic situations, the sustained action of these primary mediators through repeated stress responses can dysregulate the target systems in a process called allostatic load characterized by secondary outcomes.^24^^,^ 25 The prolonged action of glucocorticoids and glucose on mitochondrial DNA increases oxidative reactions, causing cellular dysfunction, telomere shortening, epigenetic dysregulation with altered gene expression of mitochondrial DNA, and cellular aging.^26^^,^ 27 Over time, these combined changes dysregulate the stress response target systems, whose operational parameters are modified to pathophysiological levels (above or below normal limits), as an adaptive mechanism to offset the cumulative effects of the primary mediators. As a result, the different stress response target systems manifest secondary effects such as increased metabolic mediators (insulin, glucose, total cholesterol, high-density lipoprotein, triglycerides, visceral fat deposition), cardiovascular mediators (blood pressure) and immune mediators (fibrinogen and C-reactive protein), in addition to reduced protective mediators, such as high-density lipoproteins (HDL cholesterol) and DHEA hormones.^22^^,^ 24 ^,^ 28 These mediators reach subclinical concentrations,^24^ i.e., levels above or below the expected average, indicating a load on the allostatic system (allostatic load), which is a risk condition for disease. If maintained, these effects can progressively accumulate and overload the allostatic system, triggering tertiary effects that are manifested through the emergence of cardiovascular and immunological diseases or mental and cognitive disorders.^22^^,^ 24 ^,^ 25 ^,^ 28

In summary, the effects of the stress response unfold in a structured process through a sequential chain of biological events, which starts with the adaptation of the systems to the demands of stressors, and, if prolonged, leads to multi-systemic dysregulation and culminates in disease (allostasis, allostatic state, allostatic load and allostatic overload). This process can be positively or negatively modulated by genetic, behavioral and environmental factors, biological reserves, lifestyle, and previous experiences that shape individuals’ coping and adaptation ability, i.e. resilience to stress.^29^

## CHRONIC STRESS AND ITS EFFECTS ON THE CENTRAL NERVOUS SYSTEM

The main consequence of chronic exposure to stress response mediators in the central nervous system is cellular and structural damage, primarily in the hypothalamus, cortex and hippocampus.^30^^-^ 32 In these areas, a repeated and sustained increase in the concentration of glucocorticoids triggers neurotoxic effects mediated by neuroinflammation and hyperglycemic states. The accumulation of these effects increases the production of reactive specimens and induces oxidative stress. Consequently, the mitochondrial respiratory chain decreases in activity, which curbs potential action of the membrane, impairs cellular ability to produce energy, and sensitizes neuronal apoptosis.^23^^,^ 33 In addition, neuroinflammation observed in the hippocampus of rats after exposure to chronic stress (IL-1 Beta, IL-6, TNF-alpha) also appears to result in mental health disorders and cognitive impairment because of increased proinflammatory cytokines, microglial activation and recruitment of monocytes in the caudal hippocampus, associated with temporary working memory loss and neurogenesis impairment. The rat’s immune response was a result of sympathetic activation during exposure to stressors.^34^

Although there is no direct evidence, these cellular alterations may explain the association noted in other studies between higher glucocorticoid concentration and decreased neurogenesis, dendritic arborization, and neural cell adhesion molecules (NCAM), in addition to reduced synaptic capacity and atrophy in various brain regions, including the hippocampus and cortex.^23^^,^ 24 ^,^ 30 ^,^ 31 ^,^ 35 ^,^ 36

Another effect of chronically high concentrations of glucocorticoids is hyperglycemia and decreased insulin signaling in the brain.^37^^-^ 39 Prolonged hyperglycemia alters mitochondrial morphology^37^^,^ 38 causing effects such as excessive fragmentation, oxidative stress and impaired mitochondrial DNA integrity, 22 ^,^ 40 ^,^ 41 facilitating neuronal apoptosis.^42^ Moreover, the accumulation of glucose in the blood activates immune cells, such as macrophages, which stimulate the secretion of proinflammatory cytokines (IL-1 and TNF-α). These cytokines, linked to their receptors, promote the activation of a group of kinases, which act on insulin receptors. In these receptors, densely manifested in the hypothalamus, entorhinal cortex and hippocampus,^43^ these kinases act on insulin receptor substrates, altering metabolism and insulin signaling in the brain, which promotes hyperinsulinemia and insulin resistance.^44^^,^ 45 Decreased insulin in the neurons impairs neuroplasticity, learning, and memory formation.^46^^-^ 48

From a functional point of view, one of the main effects of the accumulation of these cellular and structural damage in the hypothalamus, prefrontal cortex, and hippocampus is the progressive dysregulation of the HPA axis. Whether by direct action of the glucocorticoids or through its effect on glucose metabolism and insulin signaling, cell damage caused by these structures alters the binding sites of the MR and GR receptors in the hippocampus and prefrontal cortex, besides affecting inhibition of the HPA axis^49^ and, consequently, the negative feedback process. This maintains glucocorticoid secretion high, which produces a vicious cycle, further impairing HPA axis regulation. Another major side effect produced by the neurotoxic action of glucocorticoids at high concentrations in the hippocampus is poorer learning and memory performance.^50^ The saturation of GR receptors caused by hypercortisolism reduces long-term potentiation (LTP) - an important neurobiological substrate - and impairs memory formation. Studies have shown that excess circulating glucocorticoid due to chronic stress leads to increased activation of GR receptors. This inhibits LTP in the hippocampus^51^^-^ 53 and reduces the brain-derived neurotrophic factor (BDNF) level, which negatively affects memory performance.^54^^,^ 55

Whether these cognitive effects are temporary or permanent depends on the period of exposure to the stressor event. The harmful effects of stress are intensified during windows of vulnerability, when there is sustained action of its mediators during periods of insufficient functional biological reserves, i.e., during periods of brain development (in childhood) or during its degeneration (aging).^56^ The harmful effects of chronic stress on memory may not be permanent in adulthood, i.e., once the stressor has ceased, cortisol concentrations return to baseline levels and memory performance improves. However, the cumulative damage from exposure to stress response mediators over the lifespan produces structural changes in the brain that increase the risk of developing cognitive disorders during aging, such as AD.

## CUMULATIVE EFFECTS OF CHRONIC STRESS AND ALZHEIMER’S DISEASE

Prolonged stress conditions and the absence of adaptive coping strategies cause the accumulation of structural and functional changes in the hippocampus and prefrontal cortex, induced by the sustained action of primary mediators of stress response in the central nervous system. This constitutes a risk condition for developing cognitive impairment and dementia. The hypothesis that chronic stress may be a risk factor for AD is primarily based on the similarities between the effects of its primary mediators and the mechanisms that trigger AD (Table 1). As previously explained, chronic exposure to stress mediators induces neuroinflammation, hyperglycemic states associated with changes in glucose metabolism and insulin signaling, Aβ accumulation, and hyperphosphorylation of tau protein (Table 1). Mediated by oxidative stress, these combined changes affect neuronal functioning, altering synaptic transmission and neuroplasticity, and lead to hippocampal atrophy, causing impaired cognitive performance, especially in learning and memory (Table 1).

**Table 1:** Effects of the primary mediators of chronic stress and their similarity with the mechanisms that trigger Alzheimer's disease.

Primary mediators	Mechanisms	Effects	Relationship with
Cortisol and glucose	↑Cortisol induces hyperglycemia and insulin resistance	• Increased Ab peptide formation • Neuroinflammation, oxidative stress and cellular damage • Hyperphosphorylation of tau protein • Decreased neuroplasticity • Hippocampal atrophy and memory loss	• In individuals with MCI or AD, there is a change in the HPA axis and cortisol concentration • DM2 is associated with higher risk of AD
DHEA-S	↓DHEA-S is associated with immunological dysfunction	• Lower brain protection against Ab toxicity • Lower antioxidant defenses and vascular protection • Increased atherogenesis • Memory decline	• DHEA-S decreased in AD patients
Proinflammatory cytokines	↑IL-6 and IL-1	• Change in APP metabolism, facilitating the amyloidogenic pathway • Increased Ab deposition • Demyelination • Synaptic dysregulation and neurodegeneration	• IL-6 increased in AD patients

Ab: beta-amyloid; MCI: mild cognitive impairment; AD: Alzheimer's disease; HPA: hypothalamic-pituitary-adrenal; DM2: type 2 diabetes mellitus; DHEA-S: Dehydroepiandrosterone sulfate; IL-6: interleukin 6; IL-1: interleukin 1; APP: amyloid precursor protein.

Animal studies have shown that high concentrations of glucocorticoids (comparable with those observed in stress situations) are associated with increased Aβ peptide formation due to higher APP and β-secretase enzyme concentrations, increased phosphorylation of tau protein in the hippocampus and prefrontal cortex and, consequently, the formation of neurofibrillary tangles.^57^^,^ 58 In these studies, poorer performance in learning and memory tasks^59^^-^ 61 was also observed in animals with higher corticosterone concentrations. Peptide accumulation and the hyperphosphorylation of tau protein, induced by prolonged exposure to high corticosterone concentrations, were prevented by administering mifepristone, a glucocorticoid receptor antagonist, which strengthens the causal relationship between glucocorticoids and the neuropathology of AD.^62^ Treatment with mifepristone prevented APP cleavage by β-secretase, blocked Aβ production and, consequently, reversed cognitive deficits.^62^

**Figure 1:**
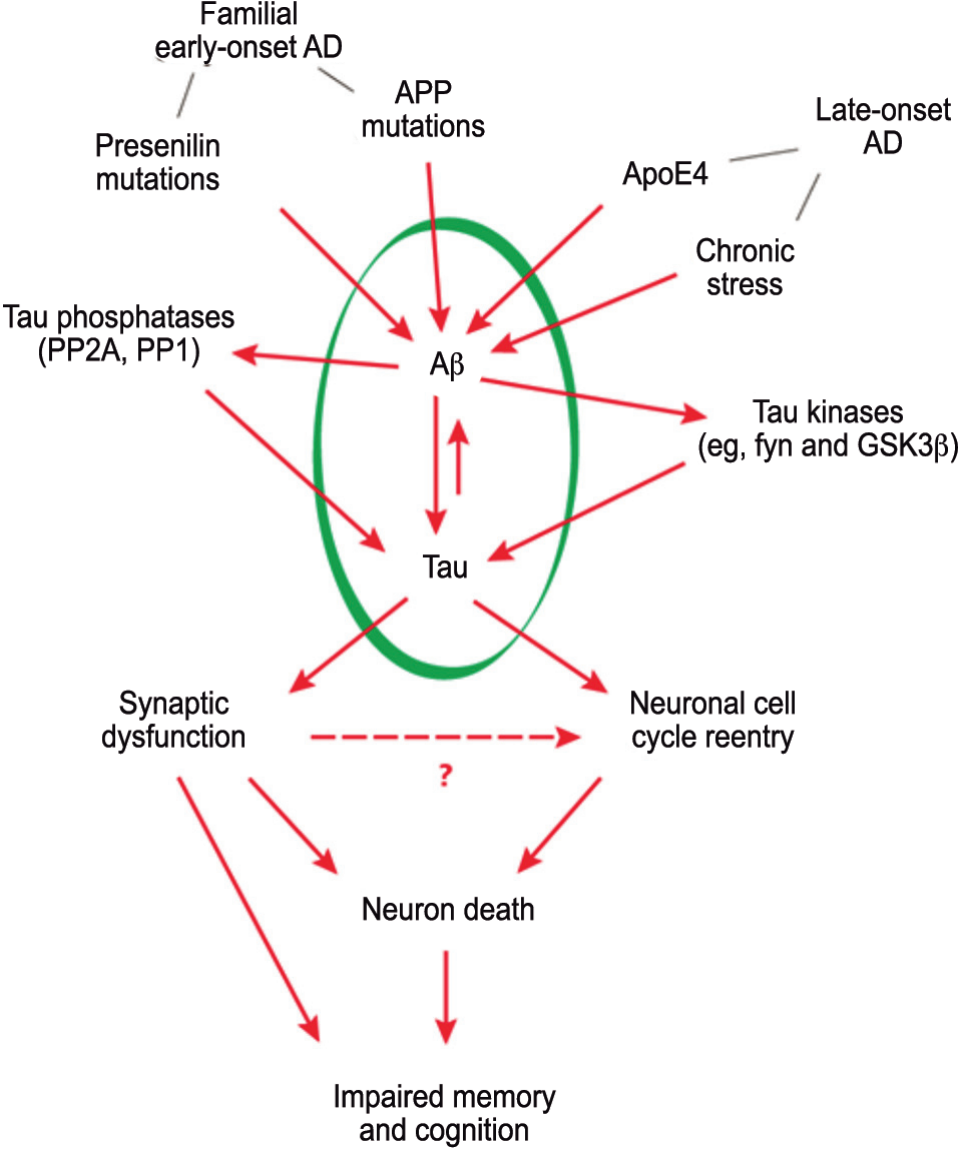
Factors associated with Amyloid-beta peptide accumulation. Chronic stress may constitute another factor that contributes to the accumulation of toxic amyloid beta in late-onset Alzheimer’s disease. Betaamyloid peptide accumulation promotes the formation of pathological tau tangles that lead to synaptic dysfunction and memory-supporting neuron death, impairing cognitive performance.

In humans, considering a continuous health-disease process, changes in cortisol concentration associated with poor cognitive performance have been observed in healthy individuals or with memory complaints in patients with MCI and AD.^63^^-^ 78 Older adults without cognitive impairment who had been exposed to intense occupational stress during adulthood had low episodic memory performance.^65^^,^ 66 A longitudinal study demonstrated that older adults with high cortisol concentrations over a period of six years had worse declarative memory performance and 14% less hippocampal volume than those with lower cortisol concentrations.^64^ Individuals with memory complaints - an important prodrome and risk factor for dementia - have higher cortisol concentrations than those not affected by this problem.^67^ Similarly, workers with high occupational stress levels at 40 years of age have a higher risk of developing MCI and AD 20 years later.^68^ Various studies have noted higher cortisol concentrations in MCI and AD patients than in cognitively healthy older adults.^69^^-^ 76 In older adults with AD^77^ or MCI,^78^ the higher the cortisol concentration, the worse the cognitive performance. Furthermore, the conversion of older adults with MCI to AD^79^ or from mild AD to moderate AD^77^ is also associated with increased cortisol concentration. As a whole, the relationship between higher cortisol and poorer cognitive performance since adulthood in individuals suffering from memory deficits^63^ - ranging from individuals with MCI to those who already have AD - strongly suggests that cortisol not only contributes to the evolution of the disease, but is also involved in the pathophysiological mechanisms that cause it.^76^^,^ 77 ^,^ 79

A large body of evidence has shown that the cumulative effects of chronic stress on AD pathology is based on the concentration of glucocorticoids (corticosterone or cortisol). However, other primary mediators of chronic stress, such as glucose, DHEA-S and proinflammatory cytokines, are also associated with AD (Table 1).

Changes in brain glucose metabolism contribute to cell degeneration and appear to be associated with the onset and progression of AD.^42^ High glucose concentrations, mediated by hypercortisolism, insulin resistance and altered glucose metabolism in the brain, are associated with Aβ accumulation and increased phosphorylation of tau protein.^80^^-^ 85 Various epidemiological studies have demonstrated an independent association between AD and type 2 diabetes mellitus (DM2). A meta-analysis found that DM2 increases the risk of developing AD by 39% regardless of the presence of cardiovascular comorbidities.^86^ Although there is no conclusive explanation for this association, one hypothesis suggests that it is mediated by moderate hypercortisolism usually present in the early stages of AD.^87^ The circadian rhythm of cortisol secretion is one of the main determinants of glycemia in humans, and high concentrations of cortisol over a long period of time may be diabetogenic due to adrenal hyperresponsiveness to ACTH.^87^ Hypercortisolism increases the risk of a pre-diabetic condition and DM2 in older adults with mild AD, decades before dementia manifests.^87^ See Table 1.

The reduced concentration of DHEA/DHEA-S inherent to aging is associated with dysfunction and activation of the immune system,^88^ increased oxidative stress^89^ and atherogenesis.^90^ DHEA is an androgen produced by the adrenal glands that acts as an antagonist against the negative effects of increased cortisol by suppressing inflammatory cytokines, improving lipid metabolism, decreasing insulin resistance, and reducing brain damage caused by oxidative stress.^91^ In a cohort study, patients with AD had lower plasma DHEA-S and DHEA levels than control volunteers.^91^^-^ 93 There is evidence that DHEA protects hippocampal cells from the toxicity produced by the accumulation of Aβ protein.^94^ Decreased circulating DHEA may also contribute to the vascular pathology observed in AD, since DHEA has antioxidant properties.^91^ By contrast, higher endogenous DHEA-S levels were independently associated with better executive function, concentration, and working memory in healthy older adults.^123^ Therefore, lower DHEA and DHEA-S concentrations undermine their neuroprotective effect, and represent a risk factor for the cumulative effects of chronic stress and progression or development of AD (Table 1).^91^^,^ 94

Regarding proinflammatory cytokines, some authors claim that AD is fundamentally an immunologically driven process, since IL-6 and IL-1 are associated with altered APP metabolism.^95^ One study used the immunohistochemistry technique on brain tissue and showed that in the early stages of AD, amyloid plaques are colocalized with acute-phase proteins and proinflammatory cytokines.^96^ Interleukin IL-1 and IL-6 have been associated with neuronal damage because of increased Aβ deposition,^97^ demyelination and neurodegeneration.^98^ IL-6 levels were high in the AD population compared to healthy older adults and inversely correlated with Mini-Mental State Examination scores.^99^ Reducing the concentration of proinflammatory cytokines could have beneficial effects on AD symptomatology.^100^ A meta-analysis demonstrated that, regardless of age, older adults with AD display higher concentrations of IL-6^99^ (Table 1).

The similarities between chronic stress and AD are not limited to the effects of their primary mediators. Secondary effects of chronic stress, represented by allostatic load biomarkers, are also associated with dementia, reinforcing the hypothesis that prolonged stress constitutes a risk factor for AD.

One of the main allostatic load biomarkers that represents the secondary outcomes of the sustained action of primary stress mediators is altered concentration of the HDL (high-density lipoprotein) fraction of cholesterol. Higher HDL concentrations are positively correlated with cognitive function.^101^^,^ 102 Some authors have reported that low HDL concentrations are associated with cognitive impairment, regardless of the presence of atherosclerotic disease.^103^ Consistent with this finding, a study conducted in middle-aged adults (55 to 61 years old) identified an association between low HDL concentrations (<40 mg/dL) and memory decline over the course of five years, even when controlling for variables known to influence cognitive performance.^104^ However, individuals with higher HDL concentrations had less cognitive impairment and improved memory performance.^105^ A meta-analysis showed a positive association between HDL and memory performance during aging.^102^ The association between low HDL concentrations and cognitive impairment is mainly because of its role in regulating the metabolism and deposition of Aβ protein,^106^ its influence on atherosclerotic disease, and its anti-inflammatory properties.^107^^-^ 109 Low apoA-I concentration (the main protein component of plasma HDL) is the main predictor of cognitive decline over the course of two years in MCI patients.^110^ In AD patients, the lower the concentration of HDL and apoA-I, the greater the severity of the disease^111^ and the risk of developing it.^112^^,^ 113

Another allostatic load biomarker, also present in AD, is a higher body mass index (BMI).^114^^-^ 116 Obesity has been associated with elevated cardiovascular and cortisol responses to acute stress.^124^ A meta-analysis showed an association between obesity and increased risk of AD.^114^ Approximately 2% of cases of AD worldwide are potentially related to obesity in middle age.^114^ A 10% reduction in the prevalence of obesity could prevent more than 66,000 cases of AD worldwide.^114^ Another study noted a U-shaped association, i.e., the two ends of BMI (low or high) were statistically and significantly associated with cognitive performance and AD risk.^116^^,^ 124 Some studies have suggested that being overweight or obese in middle age is a risk for later development of cognitive decline and dementia.^116^^,^ 125 One biological pathway hypothesized to link obesity to cognitive impairment is through leptin, a hormone mainly produced by adipocytes which suppresses appetite and regulates energy expenditure. In rodents, leptin receptor disruption is associated with impaired long-term potentiation, synaptic plasticity and spatial learning, while higher levels of leptin in the hippocampus result in decreased neurodegeneration.^126^^,^ 127 Moreover, higher plasma leptin is strongly associated with low Aβ levels in the mouse brain, supporting a protective role for the hormone in AD onset.^128^ Similarly, higher levels of leptin in humans are associated with increased hippocampal and whole brain volume and reduced incidence of AD.^129^ Conversely, decreased leptin levels in AD patients with inappropriately low weight suggests a malfunction at the hypothalamic level.^130^ With regard to cognition, one study showed leptin to have a modest protective effect against cognitive decline.^131^ Interestingly, individuals with greater adiposity and/or higher plasma leptin would be more stress-responsive.^132^

The immunological side effects of chronic stress also share similarities with AD. One meta-analysis found an association between levels of C-reactive protein (CRP) - a marker of systemic inflammation (acute phase) - and heightened risk of developing AD.^117^ Another study noted higher concentrations of high-sensitivity CRP in AD patients than in healthy individuals.^99^ Similarly, high CRP levels are associated with low memory, visuospatial impairment and low global cognitive performance.^133^^,^ 134

Finally, the allostatic load index - the estimated risk of illness due to chronic stress, which includes concentrations of primary mediators and secondary effects of stress - is related to poorer cognitive performance.^118^^-^ 120 Individuals with higher allostatic load index scores showed greater cognitive decline and mortality risk than those with lower scores.^121^^,^ 122 Furthermore, high allostatic load was associated with low total brain volume and white-matter volume and low general cognitive ability, processing speed, and knowledge in older adults.^120^

Taken together, these studies clearly demonstrate that both cognitive decline and AD are intrinsically linked to primary mediators of stress and its secondary effects manifested by allostatic load biomarkers. Given that chronic stress may be manageable, allostatic load signs allied with subjective cognitive decline may help implement preventive strategies early during adulthood to reduce the prevalence of dementia later in life. As a multisystemic effect, allostatic load may be controlled through diverse pathways including diet interventions whereby the consumption of nutrients exerting antioxidant effects may reduce the oxidative stress produced by sustained action of stress mediators.^135^^-^ 137

## CONCLUSION

Different elements produce a nonlinear and multisystemic association between the effects of the primary and secondary mediators of chronic stress and the mechanisms that trigger AD. Although acute stress is a natural and necessary response to maintain the human organism, chronic exposure to its biological mediators can cumulatively impair brain structures essential to cognitive functioning, thus representing a risk factor for cerebral aging and vulnerability to cognitive decline. The early identification in adulthood of individuals who report constant psychological stress and have altered allostatic load mediators constitutes a promising target of interventions to reduce the prevalence of dementia and contribute to successful cerebral aging. In this context, diet and nutritional status interventions can play an important preventive role.
